# Subcutaneous Granuloma Annulare in a Middle-Aged Patient: A Case Report

**DOI:** 10.7759/cureus.103506

**Published:** 2026-02-12

**Authors:** Raj Patel, Yao Liang, Padma V Chitnavis, Douglas Grider

**Affiliations:** 1 Dermatology, HCA Healthcare/University of South Florida (USF) Morsani College of Medicine, Largo, USA; 2 Medical School, Edward Via College of Osteopathic Medicine, Monroe, USA; 3 Dermatology and Mohs Surgery, Virginia Tech Carilion School of Medicine, Roanoke, USA; 4 Pathology, Carilion Roanoke Memorial Hospital, Roanoke, USA; 5 Basic Science Education, Virginia Tech Carilion School of Medicine, Roanoke, USA

**Keywords:** case report, deep granuloma annulare, necrobiosis lipoidica, palisading granuloma, rheumatoid nodule

## Abstract

Subcutaneous granuloma annulare (SGA) is a rare, benign variant of granuloma annulare, typically seen in children, which presents as asymptomatic subcutaneous nodules with normal overlying skin. The etiology remains unclear despite associations with systemic disease such as diabetes mellitus. Clinicopathologic correlation is crucial to accurate diagnosis. In this report, we present a case of a 53-year-old female patient with tender, bilateral subcutaneous nodules on her elbows. Punch biopsy confirmed the diagnosis of granuloma annulare. The primary management for her condition at the time was focused on observation and conservative care. However, secondary management with intralesional injection of triamcinolone was utilized when her lesions recurred after a window of resolution. Our case highlights an interesting manifestation of granuloma annulare and demonstrates the importance of both clinical and histological evaluation in distinguishing between similar presenting differential diagnoses. Successful treatment is heavily dependent on accurate diagnosis and patients should be monitored for complete resolution.

## Introduction

Subcutaneous granuloma annulare (SGA) is a rare, benign variant of granuloma annulare, typically seen in children, which presents as asymptomatic subcutaneous nodules with normal overlying skin [1,2]. The etiology remains unclear despite associations with systemic diseases such as diabetes mellitus and thyroid disease [3-5]. HIV and malignancy have also been found to be associated with the development of granuloma annulare [3]. Histopathologic findings demonstrate normal epidermis with palisading granulomas surrounding areas of central necrobiosis and mucin in the reticular dermis and subcutaneous tissues [1,2]. The presence of dermal mucin, which yields a bluish appearance in areas of necrobiosis on hematoxylin and eosin staining, aids in distinguishing SGA from other similarly gross-appearing lesions such as rheumatoid nodule [1]. The absence of overlying skin changes, the rarity of cases in adults, and its clinical resemblance to other subcutaneous lesions emphasizes the diagnostic challenge of SGA [2].

## Case presentation

**Figure 1:**
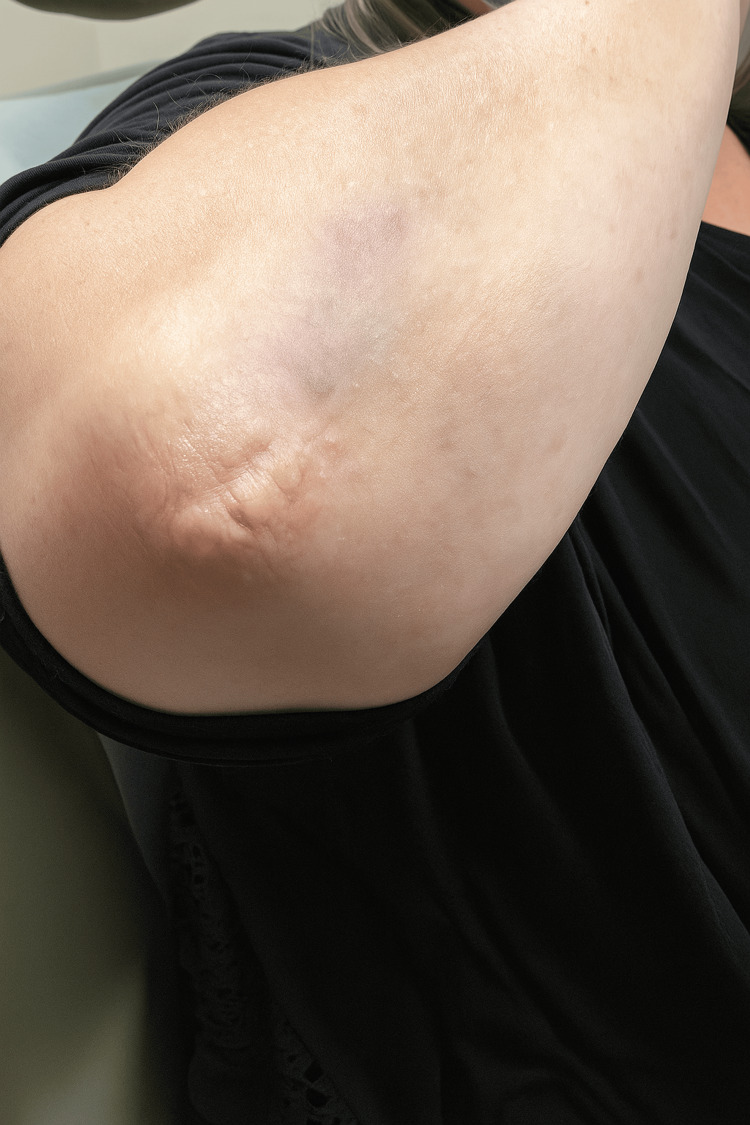
Clinical photograph revealing tender, dermal nodules, and plaques on the right elbow.

A 53-year-old female presented to the dermatology clinic for evaluation of multiple painful nodules present bilaterally on her elbows. These lesions were approximately 1 to 2 centimeters each and had been present for approximately four months. She initially addressed these lesions with over-the-counter topical corticosteroids, yielding minimal improvement. Her past medical history was notable for hypothyroidism, diabetes mellitus, and chronic gout. A detailed skin examination revealed bilateral, tender, dermal nodules and plaques on the elbows of the patient, as shown in Figure 1.

**Figure 2:**
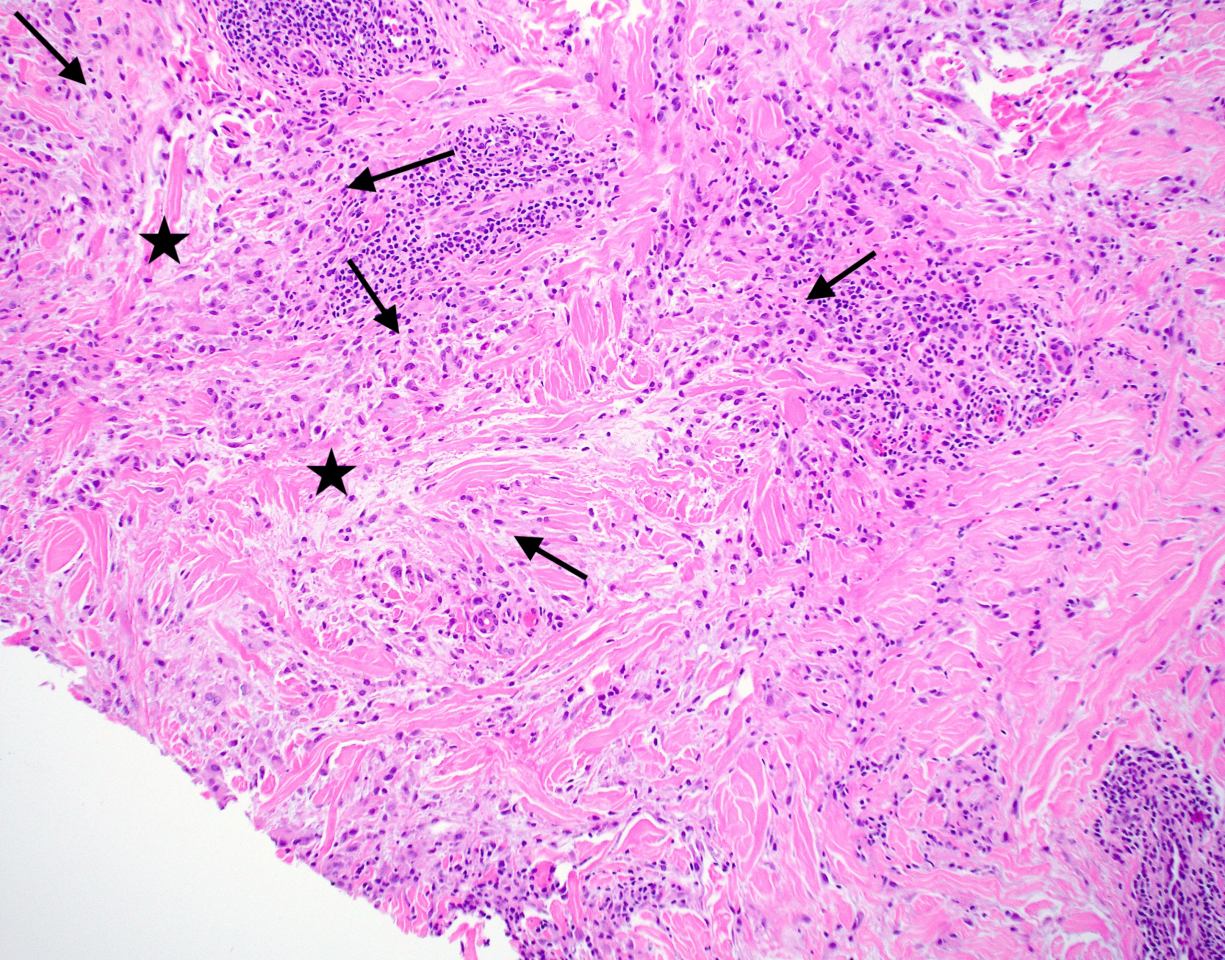
Histopathology obtained from biopsy of the right elbow demonstrating palisading histiocytes surrounding central necrobiotic material, hyaluronic acid, and mucin deposits (H&E, original magnification x 10x). Palisading granulomas surrounding central necrobiotic material are indicated by black arrows and mucin deposits noted by black stars. H&E: hematoxylin and eosin

**Figure 3:**
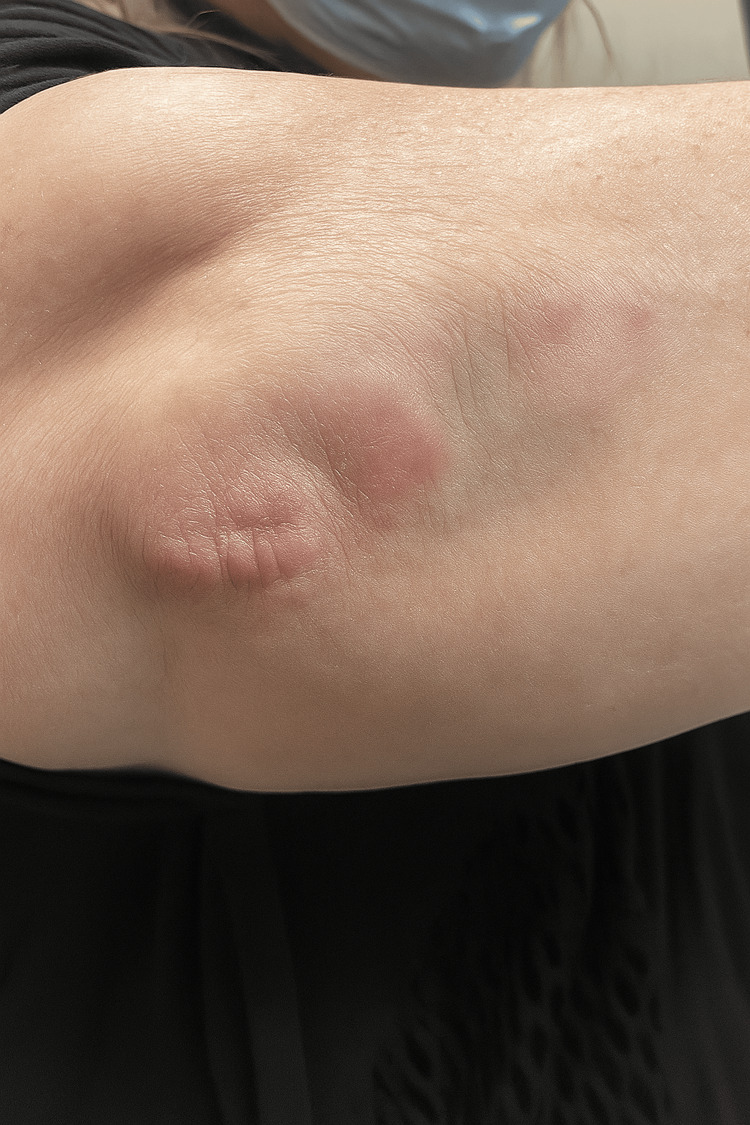
Clinical photograph revealing tender, dermal nodules, and plaques on the right elbow one month after initial presentation in the clinic.

Our initial management of the patient included performing a punch biopsy on a random nodule of the right elbow. This biopsy revealed palisading granulomas surrounding central necrobiotic material and mucin deposits, respectively noted by the black arrows and black stars shown in Figure 2. The patient presented a month later with significant improvement of the lesions without any treatment, as shown in Figure 3. However, six months later, the lesions recurred, and she was treated with intralesional triamcinolone acetonide injections at a concentration of 10 mg/mL for all the associated nodules on her bilateral elbows.

## Discussion

SGA is one of four noted subtypes of granuloma annulare [2]. SGA is commonly described as multiple painless, solitary, pinkish, nonulcerative nodules that are rapidly growing, ranging in circumference from 6 mm to 3.5 cm [6,7]. These growths are commonly localized to the extensor aspects of limbs, but have been found on the palmar side of fingers, buttocks, and scalp [6]. In a study analyzing the incidence and prevalence of granuloma annulare, a female and ethnically white predominance was observed [8]. The occurrence of SGA is rarer compared to the other variants of granuloma annulare [9]. While other studies have not specified similar results, the common report of SGA is in children less than five years old [2].

Although a direct cause is unknown, co-occurrence of physical trauma, infections, immunizations, diabetes, and dysregulation of the cell-mediated immune system have been suggested as inciting mechanisms for SGA [6,7]. In addition, shared presentations with many other disease processes create an extensive list of differential diagnoses that are difficult to discern from SGA without further workup. Thus, accurate diagnosis requires the addition of histological recognition with a clinical evaluation of the disease. Microscopic visualization of SGA yields three distinct areas: a center of homogenous necrotic collagen, surrounding palisading histiocytes, and a dense peripheral area of inflammatory cells [6]. The presence of mucin deposits in the reticular dermis and subcutaneous tissue also helps refine the diagnosis of SGA. In our case, mucin deposits were visualized and confirmed using colloidal iron staining following initial hematoxylin and eosin evaluation, further supporting this diagnosis.

Notable differential diagnoses to consider in our patient include rheumatoid arthritis, necrobiosis lipoidica, epithelioid sarcoma, and gouty tophi. While rheumatoid arthritis and epithelioid sarcoma share a similar clinical presentation to SGA, characterized by painless and well-demarcated nodules on extensor surfaces, necrobiosis lipoidica appears as well-demarcated papules that expand to yellow-brown atrophic plaques, and gouty tophi are nodules of variable sizes and shapes described as chalky [1,10,11]. Histopathological findings further emphasize differences between these differential diagnoses and SGA: rheumatoid nodules and necrobiosis lipoidica lack mucin in central necrobiotic zones; epithelioid sarcoma shows large epithelioid cells with eosinophilic cytoplasm and possible mitotic figures; and gouty tophi has an organized crystalline center with a surrounding corona zone of inflammatory cells, further encompassed by a fibrovascular zone [1,2,10,12]. Therefore, examining the gross presentation in conjunction with histopathology is the optimal method for ruling out other similar diseases when diagnosing SGA. 

The prognosis for SGA is the same as other variants of granuloma annulare: the disease course is benign and self-limiting. These lesions tend to spontaneously regress over months to years and do not require treatment [2,9]. Without any reported risk of subsequent complications, the first-line treatment involves, predominantly, observation [2]. In recurrent or symptomatic cases like ours, patients can be treated with intralesional steroid injection with concentrations selected to balance therapeutic efficacy with potential local adverse effects. Cryotherapy, phototherapy, hydroxychloroquine, TNF-alpha inhibitors, isotretinoin, imiquimod, and topical calcineurin inhibitors can also be considered in refractory cases [2,5]. As the recurrence rate for SGA varies from 40% to 80%, clinical follow-up is recommended to monitor for resolution [9].

## Conclusions

This case showcases the unique presentation of a rare variant of granuloma annulare while outlining the diagnostic approach and management. Collecting patient history and performing appropriate clinical evaluation are important as they clarify the general presentation of SGA and associated etiologies, such as diabetes mellitus. Histopathology best supplements the clinical findings, and successful treatment is heavily dependent on accurate diagnosis with monitoring for complete resolution.
